# Clinical Features at Onset and Genetic Characterization of Pediatric and Adult Patients with TNF-*α* Receptor—Associated Periodic Syndrome (TRAPS): A Series of 80 Cases from the AIDA Network

**DOI:** 10.1155/2020/8562485

**Published:** 2020-08-07

**Authors:** Carla Gaggiano, Antonio Vitale, Laura Obici, Giampaolo Merlini, Alessandra Soriano, Ombretta Viapiana, Marco Cattalini, Maria Cristina Maggio, Giuseppe Lopalco, Davide Montin, Masen Abdel Jaber, Lorenzo Dagna, Raffaele Manna, Antonella Insalaco, Matteo Piga, Francesco La Torre, Virginia Berlengiero, Viviana Gelardi, Luisa Ciarcia, Giacomo Emmi, Piero Ruscitti, Francesco Caso, Rolando Cimaz, José Hernández-Rodríguez, Paola Parronchi, Ludovico Luca Sicignano, Elena Verrecchia, Florenzo Iannone, Jurgen Sota, Salvatore Grosso, Carlo Salvarani, Bruno Frediani, Roberto Giacomelli, Maria Antonietta Mencarelli, Alessandra Renieri, Donato Rigante, Luca Cantarini

**Affiliations:** ^1^Clinical Pediatrics, Department of Molecular Medicine and Development, University of Siena, Siena, Italy; ^2^Research Center of Systemic Autoinflammatory Diseases and Behçet's Disease Clinic, Department of Medical Sciences, Surgery and Neurosciences, University of Siena, Siena, Italy; ^3^Amyloidosis Research and Treatment Center, Fondazione IRCCS Policlinico San Matteo, Pavia, Italy; ^4^Department of Internal Medicine, Arcispedale Santa Maria Nuova-IRCCS, Reggio Emilia, Italy; ^5^Rheumatology Section, Department of Medicine, University of Verona, Verona, Italy; ^6^Paediatric Clinic, University of Brescia and Spedali Civili di Brescia, Brescia, Italy; ^7^Universitary Department “Pro.S.A.M.I.”, University of Palermo, Palermo, Italy; ^8^Rheumatology Unit, Department of Emergency and Organ Transplantation, University of Bari, Bari, Italy; ^9^Division of Immunology and Rheumatology, Department of Paediatric Infectious Diseases, University of Turin, Regina Margherita Children's Hospital, Turin, Italy; ^10^Rheumatology Unit, Santa Chiara Hospital, Trento, Italy; ^11^Unit of Immunology, Rheumatology, Allergy and Rare Diseases (UnIRAR), IRCCS San Raffaele Scientific Institute, Milan, Italy; ^12^Department of Internal Medicine, Vita-Salute San Raffaele University, Milan, Italy; ^13^Periodic Fever Research Center, Institute of Internal Medicine, Catholic University of the Sacred Heart, Fondazione Policlinico A. Gemelli, Rome, Italy; ^14^Division of Rheumatology, Department of Pediatric Medicine, Bambino Gesù Children's Hospital, IRCCS, Rome, Italy; ^15^Rheumatology Unit, Department of Medical Sciences, University and AOU of Cagliari, Cagliari, Italy; ^16^Clinical Pediatrics, University of Bari, Bari, Italy; ^17^Department of Experimental and Clinical Medicine, University of Firenze, Florence, Italy; ^18^Rheumatology Unit, Department of Biotechnological and Applied Clinical Sciences, University of L'Aquila, Italy; ^19^Rheumatology Unit, Department of Clinical Medicine and Surgery, School of Medicine and Surgery, University Federico II, Naples, Italy; ^20^Department of Clinical Sciences and Community Health, University of Milan, Milan, Italy; ^21^Rheumatology Unit, A. Meyer Children's Hospital, Florence, Italy; ^22^Vasculitis Research Unit and Autoinflammatory Diseases Clinical Unit, Department of Autoimmune Diseases, Hospital Clinic of Barcelona, IDIBAPS, University of Barcelona, Barcelona, Spain; ^23^Genetica Medica, Azienda Ospedaliera Universitaria Senese, Siena, Italy; ^24^Medical Genetics, University of Siena, Siena, Italy; ^25^Institute of Pediatrics, Periodic Fever Research Center, Università Cattolica Sacro Cuore, Fondazione Policlinico A. Gemelli, IRCCS, Rome, Italy

## Abstract

This study explores demographic, clinical, and therapeutic features of tumor necrosis factor receptor-associated periodic syndrome (TRAPS) in a cohort of 80 patients recruited from 19 Italian referral Centers. Patients' data were collected retrospectively and then analyzed according to age groups (disease onset before or after 16 years) and genotype (high penetrance (HP) and low penetrance (LP) *TNFRSF1A* gene variants). Pediatric- and adult-onset were reported, respectively, in 44 and 36 patients; HP and LP variants were found, respectively, in 32 and 44 cases. A positive family history for recurrent fever was reported more frequently in the pediatric group than in the adult group (*p* < 0.05). With reference to clinical features during attacks, pericarditis and myalgia were reported more frequently in the context of adult-onset disease than in the pediatric age (with *p* < 0.01 and *p* < 0.05, respectively), while abdominal pain was present in 84% of children and in 25% of adults (*p* < 0.01). Abdominal pain was significantly associated also to the presence of HP mutations (*p* < 0.01), while oral aphthosis was more frequently found in the LP variant group (*p* < 0.05). Systemic amyloidosis occurred in 25% of subjects carrying HP variants. As concerns laboratory features, HP mutations were significantly associated to higher ESR values (*p* < 0.01) and to the persistence of steadily elevated inflammatory markers during asymptomatic periods (*p* < 0.05). The presence of mutations involving a cysteine residue, abdominal pain, and lymphadenopathy during flares significantly correlated with the risk of developing amyloidosis and renal impairment. Conversely, the administration of colchicine negatively correlated to the development of pathologic proteinuria (*p* < 0.05). Both NSAIDs and colchicine were used as monotherapy more frequently in the LP group compared to the HP group (*p* < 0.01). Biologic agents were prescribed to 49 (61%) patients; R92Q subjects were more frequently on NSAIDs monotherapy than other patients (*p* < 0.01); nevertheless, they required biologic therapy in 53.1% of cases. At disease onset, the latest classification criteria for TRAPS were fulfilled by 64/80 (80%) patients (clinical plus genetic items) and 46/80 (57.5%) patients (clinical items only). No statistically significant differences were found in the sensitivity of the classification criteria according to age at onset and according to genotype (*p* < 0.05). This study describes one of the widest cohorts of TRAPS patients in the literature, suggesting that the clinical expression of this syndrome is more influenced by the penetrance of the mutation rather than by the age at onset itself. Given the high phenotypic heterogeneity of the disease, a definite diagnosis should rely on both accurate working clinical assessment and complementary genotype.

## 1. Background

Tumor necrosis factor receptor-associated periodic syndrome (TRAPS) is one of the most common autosomal dominant autoinflammatory diseases and is caused by mutations in the tumor necrosis factor receptor super family member 1A (*TNFRSF1A*) gene on chromosome 12p13. Originally identified in northern Europe and French-Canadian ancestry families during the last decades of the XX century, it has been recognized thereafter mainly in Caucasians, but also in patients from Africa and Asia, with an estimated prevalence of about one per million people [1]. The incidence of TRAPS is very low; it has been calculated as 5.6 cases per 10^7^ person-years for the period 2003–06 in German children, which corresponds to 6–10 newly diagnosed young patients per year [2]. Nevertheless, like other rare conditions, this multifaceted disease is potentially both underdiagnosed and underreported.

Among the expanding spectrum of hereditary recurrent fevers, TRAPS remains one of the most variable as concerns clinical features, age at onset, and disease severity, ranging from the mildest phenotype often associated to low-penetrance (LP) genetic variants to a severely disabling condition in patients carrying structural high-penetrance (HP) variants; in the latter group, systemic amyloidosis represents the most threatening complication of the syndrome, affecting 10-15% of untreated subjects [3, 4]. Patients may display a relapsing-remitting course, with prolonged high-grade fever attacks accompanied by a systemic inflammatory reaction and localized flogosis of any potential site, with skin, muscles, abdomen, eye, joints, serous membranes, and lymph nodes being the most frequently targeted [5]. Otherwise, patients may exhibit a chronic course, characterized by symptomatic intervals between fever attacks and/or inflammatory markers steadily elevated.

Actually, neither functional tests nor diagnostic criteria are available to identify the disease in the clinical setting, and TRAPS diagnosis relies on the detection of a pathogenic variant of the *TNFRSF1A* gene. Currently, more than 160 gene mutations have been found, and 99 of them are classified as pathogenic or likely pathogenic, according to *Infevers* database (*Infevers*: an online database for autoinflammatory mutations. Copyright. Available at https://infevers.umai-montpellier.fr/ Accessed 2019.12.22) [6]. The majority of these disease-associated mutations are identified in exons 2, 3, and 4, encoding the extracellular domain of TNF receptor. The uncertainty regarding the pathogenic role of some LP mutations, such as R92Q and P46L, whose estimated allele frequency is 10% and 2% in West African and Caucasian population, respectively, further complicates the diagnostic challenge, since many carriers do not manifest any symptom, while other ones develop a mild-moderate phenotype with specific clinical features [7, 8]. Classification criteria for TRAPS have been recently developed and validated on the basis of *Eurofever* Registry by the Paediatric Rheumatology International Trials Organisation (PRINTO) [9]. Moreover, specific clinimetric tools have been recently proposed to assess disease activity and disease-induced systemic organ damage. In particular, the autoinflammatory disease activity index (AIDAI) is used in clinical practice for measuring disease activity during a 1-month period according to the presence or absence of selected inflammatory manifestations reported day by day [10]; the autoinflammatory disease damage index (ADDI) is used to establish chronic organ damage owing to disease activity over time [11].

Few studies in the literature compared clinical features according to the age of disease onset [1, 3, 12], and no data at all are available about the performance of the new TRAPS classification score in different age groups and compared to the preexisting Eurofever score [13]. This multicenter study was designed to define demographic, clinical, and therapeutic features of a large cohort of TRAPS patients in Italy, distinguished according to different age at onset and genetic variants, on the basis of the nation-wide collaborative experience of the Autoinflammatory Disease Alliance (AIDA) Network and the Working Group for Autoinflammatory Disease of the Italian Society of Rheumatology.

## 2. Patients and Methods

This retrospective observational multicenter study recruited 80 patients from nineteen Italian rheumatology and pediatric rheumatology tertiary referral Centers participating to the AIDA Network and to the Working Group for Autoinflammatory Disease of the Italian Society of Rheumatology. Patients were included in the study if TRAPS was diagnosed by the referring physician on the basis of consistent clinical history and genotype. A structured form was sent to local investigators, collecting demographic, clinical, therapeutic, and clinimetric data from clinical charts. Provisional Eurofever score for TRAPS according to Federici et al. [13] was calculated for every patient by the referring physician; classification scores for TRAPS, FMF, mevalonate kinase deficiency (MKD), and cryopyrin-associated periodic syndromes (CAPS) were subsequently derived by the authors on the basis of available data, according to Gattorno et al. [9].

According to criteria used in previous studies, patients aged <16 and ≥16 years at disease onset were considered to have pediatric and adult disease onset, respectively [2, 3, 14, 15]. In order to distinguish patients according to genotype, we divided the cohort into two main groups: patients carrying *TNFRSF1A* HP mutations (HP group) and patients carrying *TNFRSF1A* LP mutations (LP group). Three patients with a convincing clinical phenotype had no mutations detected using Sanger sequencing; however, somatic mosaicism could not be formally excluded at the time of enrolment. These patients, along with a fourth patient carrying a mutation with unknown penetrance, were excluded when the statistical analysis was performed to assess features according to genetic groups. Phenotypical characteristics of specific genetic subgroups (R92Q; mutations involving a cysteine residue; HP mutations not involving cysteine residues) were also explored.

Regarding response to treatment, “complete response” was defined as complete control of clinical and laboratory manifestations; “partial response” was meant as: (i) a decrease in clinical severity of flares corresponding to a less severe systemic inflammation during disease attacks; (ii) a patient-reported improvement in clinical manifestations during flares for relapsing-remitting disease courses or outside of flares for chronic cases. The response of patients not meeting these criteria was defined as “complete inefficacy”.

The study protocol was conformed to the tenets of the Declaration of Helsinki and was approved by the local Ethics Committee of the University of Siena (Reference No.14951). Written informed consent for using clinical data for research purposes was obtained according to the local Institutional review board guidelines.

### 2.1. Aims of the Study

The primary aim of the study was to describe demographic, clinical, and therapeutic features of TRAPS according to age at disease onset in a large cohort of patients.

Secondary aims were (I) to describe demographic, clinical and therapeutic features of TRAPS according to the penetrance of *TNFRSF1A* variants (HP variants group versus LP variants group) and specific genotype subgroups (R92Q; HP mutations involving a cysteine residue; HP mutations not involving cysteine residues); (II) to evaluate how TRAPS classification criteria [9, 13] work across different age and genetic groups; (III) to explore any associations between clinical/laboratory features of the whole cohort of patients at disease onset and prognostic indicators including the occurrence of systemic amyloidosis at the time of diagnosis and pathologic proteinuria.

### 2.2. Statistical Analysis

Data were analyzed using IBM SPSS Statistics. Descriptive statistics included sample sizes, mean and standard deviation (SD), or median and interquartile range (IQR), as appropriate. Shapiro–Wilk test was used to assess the normality distribution of data. Categorical variables were analyzed using 2 × 2 contingency tables with Fisher's exact test, while analysis of means was investigated through Mann–Whitney *U* test or unpaired samples *T*-test, as appropriate. Correlations between clinical features and prognostic indicators were performed employing Kendall's tau-b test. Binary logistic regression was performed to detect potential factors predictive of the development of amyloidosis or pathologic proteinuria. Logistic or nominal regression was applied to find any impact of the treatment duration with biologic agents or the duration of follow-up at the last visit on the following outcomes: discontinuation of biologic therapy, dose adjustments of biologic agents, occurrence of adverse events during biologic treatment, and the ADDI score recorded at the end of follow-up. The threshold for statistical significance was set to *p* < 0.05, and all *p* values were two-sided.

## 3. Results

### 3.1. Demographic and Genotypical Characteristics

Demographic characteristics of TRAPS patients in the whole cohort and in different age at onset and genetic groups are summarized in Table 1. Caucasian ethnicity was the most represented (92.5%), while Arabic, Afro-American, and Jewish ethnicities accounted for 2.5%, 2.5%, and 1.3%, respectively. Forty-four individuals (55%) had pediatric-onset disease and 36 (45%) had adult-onset disease. Mean diagnostic delay was 23.4 ± 16 years (range 1.0-44.0) in the pediatric-onset group and 15.6 ± 11.6 years (range 2.0-31.0) in the adult-onset group (*p* < 0.001). Mean disease duration at last follow-up visit was 33.8 years in the pediatric-onset group and 14.2 years in the adult-onset group (*p* = 0.002).

**Figure 1:**
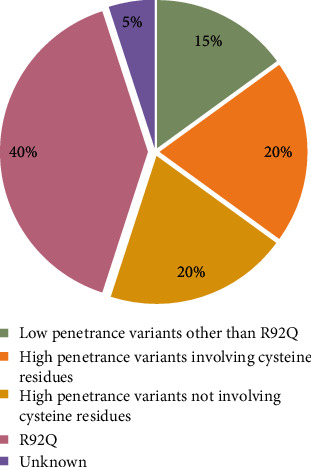
Genotypical characterization of TRAPS patients.

Details about the genotypical characterization of the cohort are summarized in Figure 1 and Table 2. In particular, *TNFRSF1A* HP mutations were detected in 32 patients (40%) and LP mutations in 44 patients (55%). The median age at onset was 5.0 (*IQR* = 14.1) years (range 1.0-49.0) among HP carriers and 16.0 (*IQR* = 26.5) years (range 1.0-48.0) among LP carriers, with *p* = 0.01. In the HP group, pediatric-onset disease occurred in 94% of cases among patients carrying cysteine substitutions and in 56% of cases among patients with HP mutations not involving cysteine residues (*p* = 0.01). Median diagnostic delay was significantly longer in the group of patients carrying *TNFRSF1A* HP mutations (30.0 years, *IQR* = 26.0; min 1.0, max 60.0) than in the LP group (7.0 years, *IQR* = 15.0; min 1.0, max 47.0) with *p* < 0.001. No statistically significant differences were found as concerns ethnicity and sex predominance.

### 3.2. Clinical Features at Disease Onset

A familial history of recurrent fever was present in about 38% of cases. The disease displayed a relapsing-remitting course in 59 patients (74%) and a chronic course in 21 cases (26%). Before starting biologic therapy 43% of patients had 2-6 attacks per year, 34% had 6-12 attacks per year, 15% more than 12 attacks per year; 8% of subjects had less than 2 attacks per year. The mean duration of fever attacks was 13 days, ranging from 1.5 to 30 days. Musculoskeletal symptoms were the most common clinical features accompanying fever: arthralgia and myalgia were reported in 70% and 66% of patients, respectively, while arthritis was less common (34%). Abdominal pain and chest pain were presented by 58% and 34% of individuals. Other common clinical features were skin rash in 41% of cases (urticarial in 27.5%, erythematous in 6.3%, maculopapular in 5%, erythema nodosum in 2.5% and petechial rash in 1.3% of the cohort) and lymph nodes enlargement in 38% of cases, while other manifestations were less frequently reported (pharyngitis 30%, oral aphthosis 25%, pericarditis 22.5%, fatigue 18.8%, conjunctivitis 17.5%, periorbital edema 17.5%, liver and/or spleen enlargement 11.3%, pleurisy 10%, headache 10%, diarrhea 3.8%, vomiting 2.5%, tinnitus 1.3% and scleritis 1.3%). In detail, pericarditis was reported in 18 patients, with a mean age at disease onset of 29 years (range 4-59 years). In 6 cases, a concurrent involvement of the pleural serosa was detected; moreover, arthralgia, myalgia, and arthritis were the most frequent accompanying manifestations (in 14, 10, and 8 out of 18 patients, respectively). Systemic amyloidosis and pathologic proteinuria were present, respectively, in 8 and 9 patients out of 80 at diagnosis.

**Figure 2:**
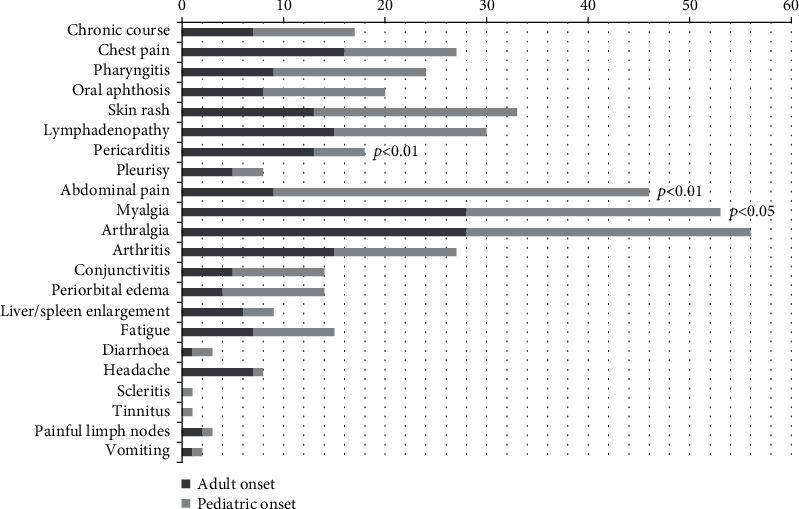
Clinical characteristics of TRAPS patients at disease onset, according to age at onset.

Figure 2 displays the clinical characteristics of TRAPS patients at disease onset, according to age at onset. A familial history of recurrent fever was present in 52% of pediatric-onset cases and in 22% of adult-onset cases (*p* = 0.02). Among clinical features during attacks, pericarditis and myalgia were reported more frequently in the context of adult-onset disease than in the pediatric age (with *p* = 0.008 and *p* = 0.04, respectively) while abdominal pain was present in 84% of children and in 25% of adults (*p* < 0.001). No other statistically significant differences were found between the two groups as concerns clinical presentation and laboratory markers, namely mean C-reactive protein (CRP) and erythrocyte sedimentation rate (ESR) measured during fever attacks and mean serum amyloid A protein (SAA) and mean 24-hours proteinuria measured outside of fever episodes.

**Figure 3:**
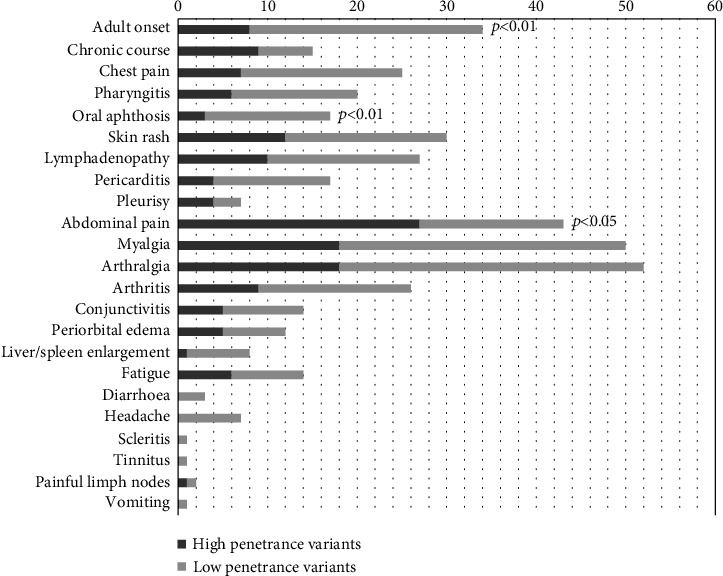
Clinical characteristics of TRAPS patients at disease onset, according to the penetrance of *TNFRSF1A* mutations.

Figure 3 displays the clinical characteristics of TRAPS patients at disease onset, according to the penetrance of *TNFRSF1A* mutations. Subjects carrying HP mutations had a consistent familial history in 56% of cases, while in the LP group the frequency was 25% (*p* = 0.04). Regarding clinical manifestations, abdominal pain was significantly more frequent in patients with HP mutations (84% versus 36%, *p* < 0.001), while oral aphthosis was more frequently reported in the LP variant group (32% versus 9%, *p* = 0.02). Moreover, among HP variants group, lymphadenopathy was significantly more frequent in the non-cysteine group than in the cysteine group (*p* = 0.02). The difference in the occurrence of systemic amyloidosis between the two groups was highly significant (25% of HP variants patients versus 0% of LP variants patients, *p* = 0.002). As concerns laboratory features, patients with HP mutations had higher ESR than that observed in LP group (86.6 ± 37.4 versus 60.0 ± 27.2 mm/h, *p* = 0.003); inflammatory markers steadily increased during intervals between attacks were more commonly found in the HP than in the LP variants group (28% and 9% of cases respectively, *p* = 0.04).

### 3.3. Clinimetrics and Classification Criteria

At disease onset, median AIDAI and ADDI scores were 10 (*IQR* = 13) (min. 0; max. 120) and 1 (*IQR* = 2) (min. 0; max. 12), respectively, with no statistically significant difference between groups. AIDAI score at the start of biologic therapy was stable in 33% of patients, increased in 9% of patients, and decreased in 16% of cases, without any significant difference between groups. AIDAI score decreased after 3, 6, and 12 months since the start of biologic therapy and at last follow-up visit in 95.5%, 88.1%, 92.5%, and 88.4% of patients, respectively.

The edition of the Eurofever score by Federici et al. [13] was fulfilled by 57/80 patients (71.3%). The difference between the sensitivity of the score in the HP variant group (93.8%) and in the LP variant group (56.8%) was highly significant (*p* < 0.001). The latest TRAPS classification criteria proposed by Gattorno et al. for patients carrying *TNFRSF1A* gene variants [9] were satisfied at disease onset by 64 out of 80 patients (80%); TRAPS classification criteria purely based on clinical features (and no reference to genetic findings) [9] were fulfilled by 46 out of 80 patients (57.5%). No statistically significant differences were found in the sensitivity of the classification criteria according to age at onset and according to genotype, as displayed in Table 3 along with percentages of the fulfillment of the two different editions of the Eurofever scores.

### 3.4. Prognostic Data

As concerns prognostic data, mutations involving a cysteine residue and the presence of abdominal pain during attacks at disease onset significantly correlated with the development of pathologic proteinuria (*p* = 0.001 and *p* = 0.04, respectively); lymphadenopathy during attacks significantly correlated with the development of amyloidosis (*p* = 0.018); the discontinuation of biologic treatment because of secondary inefficacy was significantly correlated with the development of amyloidosis and pathologicproteinuria (*p* = 0.03 in both cases), while the use of colchicine was negatively correlated with the development of pathologic proteinuria (*p* = 0.03). No other significant correlations were found between other clinical, laboratory and therapeutic data, and prognostic measures. Binary logistic regression detected lymphadenopathy during febrile episodes as the only variable associated with the development of amyloidosis (OR = 6.14 [1.14-33.07], *p* = 0.04), and the presence of mutations affecting cysteine residues as the only variable associated with the development of pathologic proteinuria (OR = 6.82 [1.58-29.46], *p* = 0.01).

### 3.5. Characteristics of Patients Carrying R92Q Variant

**Figure 4:**
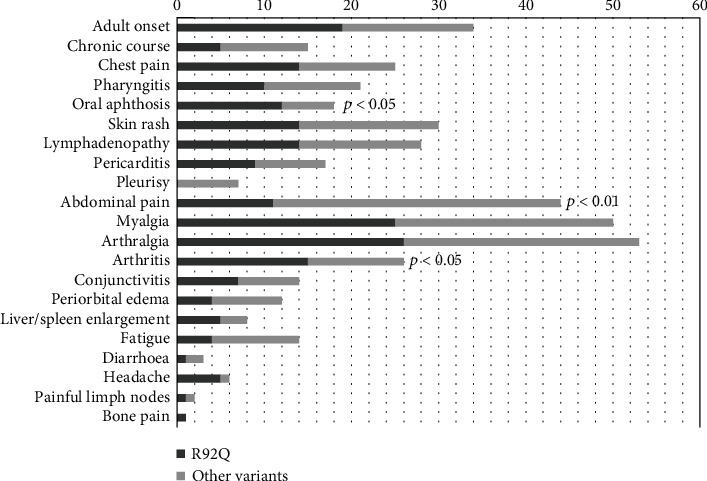
Clinical characteristics of patients carrying R92Q variant at disease onset, compared with patients carrying other *TNFRSF1A* variants.

The distribution of clinical manifestations across the R92Q mutation group (*n* = 32) is displayed in Figure 4. They had a median age at onset of 5 (*IQR* = 15) years, with disease onset occurring in the pediatric age and in the adult age in 41% and 59% of cases, respectively. The mean duration of attacks was 12 days, and 55% of patients had more than 6 attacks per year. Oral aphthosis and arthritis were more frequently reported among R92Q patients (*p* = 0.04 in both cases), while abdominal pain was less commonly experienced (*p* = 0.001), and no cases of amyloidosis were reported at all in this subgroup. Mean ESR during episodes was significantly lower than ESR recorded among patients with different mutations (49 versus 74 mm/h, *p* = 0.007). The last Eurofever TRAPS classification criteria were met in 53.3% (17/32) of cases when only clinical features were included and 81.3% (26/32 patients) when considering genotype. R92Q patients were more frequently on nonsteroidal anti-inflammatory drugs (NSAIDs) monotherapy than other patients (78.1% versus 37.5% of cases, *p* < 0.001); biologic therapy was administered in 53.1% of cases (13 anakinra, 2 canakinumab, 2 etanercept).

### 3.6. Therapeutic Data

Regarding treatment, NSAIDs have been administered as monotherapy in about half of TRAPS patients (53.8%), on a daily regimen (17.5%), on demand (27.5%), or with unknown regimen (8.8%). About 16% of them reported complete response to NSAIDs, 58% partial response, 19% complete inefficacy, 7% unknown response. NSAIDs were used as monotherapy more frequently in the group of adult-onset disease than in the group of pediatric-onset (64% versus 46%, *p* < 0.001), and more frequently in the LP mutations group compared to the HP variants group (71% versus 31%, *p* = 0.001). There were no significant differences between groups as concerns therapeutic regimens and clinical response to NSAIDs.

Glucocorticoids (GC) were administered as monotherapy in 60 TRAPS patients (75%), on a daily regimen (36.3%), on demand (32.5%), or with unknown regimen (6.3%). Daily dose ranged from 5 to 150 mg/day (mean dose = 35 mg/day) and from 0.6 to 2.5 mg/kg/day (mean dose = 1.2 mg/kg/day). Complete response to CS as monotherapy was reported by 53% of patients, partial response in 28% and complete inefficacy in 12% (data not available in 7% of cases). There were no statistical differences between groups as concerns therapeutic regimen and clinical response to GC.

Colchicine was administered as monotherapy in 31 TRAPS patients (38.8%). About 10% of them reported complete response to colchicine, 45% partial response, 23% complete inefficacy (data not available in 22% of cases). Colchicine was used as monotherapy more frequently in the LP mutations group compared to the HP variants group (50% versus 25%, *p* = 0.002). There were no other significant differences between groups as concerns employment and clinical response to colchicine.

Biologic agents were prescribed to 49 (61%) patients. In detail, any biologic course was prescribed to 20 (56%) patients of the adult-onset group, 29 (66%) patients of the pediatric-onset group, 23 (72%) of the HP group, and 24 (55%) of the LP group. No statistically significant differences emerged between groups about the frequency of biological therapy administration. Interleukin-1 (IL-1) inhibitors were chosen as first-line biologic agent in 86% of cases (anakinra in 28 subjects, canakinumab in 14 subjects); the remaining patients were treated with a TNF inhibitor as first-line biologic drug (adalimumab, infliximab and etanercept in 1, 1 and 5 cases, respectively). The majority of patients were initially treated with standard posology (88%), while the initial dose of the biologic agent was higher in 4 cases—treated with anakinra (1), canakinumab (2), or adalimumab (1)—and lower in 1 case undergoing anakinra. In 18 (37%) cases, the initial posology was adjusted during follow-up, by increasing or decreasing the dose (in 2 and 4 cases, respectively) or by shortening or increasing intervals between administrations (in 2 and 10 cases, respectively). Mean disease duration at the start of biologic therapy was 20 years (min. 1; max. 65), and the mean duration of biologic therapy was 38 months (min. 1; max. 120). About 73% of patients reported complete response to biologic agents, 12% partial response, and 6% complete inefficacy (data not available in 9% of cases). Fifteen (31%) subjects discontinued the therapy due to primary inefficacy (*n* = 3, 6%), secondary inefficacy (*n* = 7, 14%), adverse events (*n* = 2, 4%), lack of compliance (*n* = 2, 4%), or other reasons (*n* = 5, 10%). Eight patients manifested adverse events during biologic treatment (16%): 3 subjects had injection site reactions to anakinra, 1 had chest pain and 1 amnesia with anakinra, 1 subject had recurrent low urinary tract infections and 1 varicella-zoster virus infection with canakinumab, and 1 subject had a paradoxical inflammatory reaction to infliximab.

Logistic regression analysis did not find any relation between the duration of biologic therapy or the duration of follow-up and discontinuation of therapy, dose adjustments, and occurrence of adverse events.

**Figure 5:**
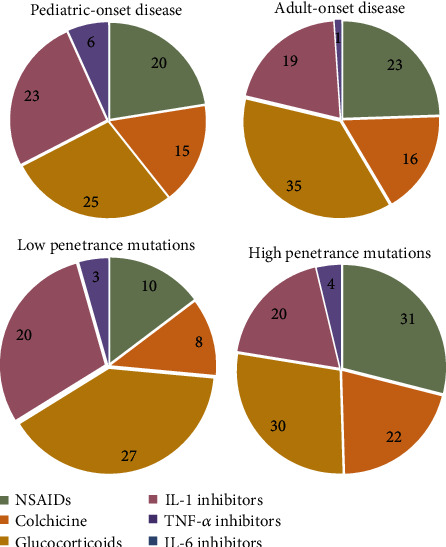
Therapeutic regimens in TRAPS patients, according to age at onset and genotype. Nonsteroidal anti-inflammatory drugs, colchicine, and glucocorticoids shall be considered as monotherapy. Only the first biologic line of treatment is shown in the charts. List of abbreviations: NSAIDs: nonsteroidal anti-inflammatory drugs.

The frequency of therapeutic regimens in TRAPS patients, according to age at onset and genotype is displayed in Figure 5.

## 4. Discussion

The present study outlines the characteristics of a large cohort of TRAPS patients resulting from a positive nation-wide collaborative research experience in the field of autoinflammatory diseases. The size of this series gave us the opportunity to explore how this extremely rare disease shows up to the attention of both pediatric and adult services, a matter of the utmost relevance since the diagnosis of TRAPS relies on the detection of a confirmative mutation in the causative gene through specific tests of limited availability and significant expense.

Although disease onset is known to occur usually around the age of four, a significant variability emerges from the literature, ranging from the first weeks of life to the sixties [16–18]. In our cohort, the median age at onset was eight years, but disease presented itself in adulthood in almost half of patients (range 1-59 years). The evidence that an earlier onset of symptoms is accompanied by the detection of HP gene variants is well known in the literature [7, 8], and this data is further validated in this series; interestingly, we found that mutations involving cysteine residues accounted for the earlier onset in the HP variant group (94% pediatric-onset), while patients with HP mutations not involving cysteine residues (T50M, S59P, c.472+1G>A, R53G, c.586_612del27, and Y103_R104DEL) had pediatric-onset only in 56% of cases. Data previously reported from the Eurofever registry showed that adult patients with disease onset in childhood carried a single variant involving T50M or a cysteine residue in 51% of cases, while patients with an adult-onset carried them in 23% of cases, and the difference was statistically significant [1]. Papa et al. reported a mean age at onset of 9.4 years in patients carrying T50M, S59P, or del130-104 variants and a mean age at onset of 2.6 years in patients carrying the cysteine mutations C43R, C43Y, C52Y, and C55Y [17]. Such early onset disease may be explained by the crucial role of the cysteine-rich domains for the preservation of the three-dimensional conformation of the extracellular part of the TNF receptor, which relies on the presence of intramolecular Cys-Cys disulfide bonds. Thus, a substitution at a cysteine residue results in a significant disruption of the structure of the protein, with a relevant pathophysiologic impact on the course of the disease [19]. Diagnostic delay was considerable both in children and in adults, as reported also in patients from the Eurofever registry [1]. More in detail, the diagnostic delay was significantly more pronounced in the case of childhood-onset and in patients carrying HP gene variants than in adult-onset disease. Given the fact that the wide majority of patients were adults at the time of data collection (94%), with a median age of 44 years (range 9-77 years), we assume that such discrepancy reflects the improving knowledge of TRAPS in the last decades. Actually, pediatric-onset patients manifested the disease far back in time compared to adult-onset patients; thus, diagnostic capabilities, only recently improved, have brought about a reduction in the diagnostic delay especially among patients with adult-onset disease.

With regard to the clinical spectrum of the disease, our group previously observed that adult patients may both exhibit a phenotype that mimics other autoinflammatory disorders characterized by shorter inflammatory attacks, such as familial Mediterranean fever, and present with oligosymptomatic disease or atypical manifestations including isolated myocarditis and pericarditis [20]. Consequently, adult-onset TRAPS may frequently lead to misdiagnosis and improper management [8, 18, 21]. Data from the present study confirm the higher frequency of pericarditis in the context of adult-onset disease and show a higher frequency of myalgia in this subgroup as well. More in detail, in our cohort, eighteen patients had pericarditis at onset, with concurrent involvement of other serousal membranes in six cases. In this subgroup, the mean age at onset was 29 years, and mild musculoskeletal symptoms (arthralgia and myalgia) were reported more frequently than other complaints. These findings are consistent with data recently published from the Eurofever registry by ter Haar et al. on patients with undefined AIDs [22]: arthralgia, myalgia, and abdominal pain were often reported together with pericarditis, suggesting that the presence of this combination could be a clue for suspecting specific AIDs in patients with otherwise idiopathic pericarditis. Indeed, the extension of genetic analysis allowed the identification of the R92Q mutation in two out of eight cases.

On the other hand, cervical lymphadenopathy, ocular manifestations, and abdominal pain seem to be more frequently complained by children according to current literature [1, 8]. In particular, data from the Eurofever cohort showed that abdominal pain at disease onset was similarly complained by children and adults with pediatric-onset disease, while its frequency was significantly lower in the adult-onset group [1]. In line with these findings, abdominal pain was the only clinical feature specifically associated with the pediatric age at onset in our cohort. Similar data can be derived from different case series reporting data from pediatric and adult-onset TRAPS: Dodé et al., basing on 28 unrelated patients, reported abdominal pain in 86.6% of pediatric-onset patients and 46.2% of adult-onset cases [23]; Federici et al. published 19 TRAPS patients, with abdominal involvement reported in 91.6% of pediatric-onset disease [24]. On the contrary, in a large cohort from NIH-Nottingham [5], the frequency of abdominal pain was less remarkable between pediatric and adult-onset TRAPS (92.3% versus 72.7%); nevertheless, the majority of patients presenting with abdominal pain had HP variants, which are *per se* strongly associated to the presence of abdominal pain at onset, as also highlighted in the present study.

As expected, in the present study, patients with HP mutations had more frequently a family history of recurrent fever and higher levels of inflammatory markers with the tendency to persist above the normal range also during intercritic periods. Systemic amyloidosis at the time of diagnosis affected only individuals carrying HP variants; noteworthy, the presence of cysteine-mutations, abdominal pain, or lymphadenopathy during attacks at disease onset represented risk factors for the development of amyloidosis and/or pathologic proteinuria. These findings agree with Lane et al., who reported abdominal pain as the most represented symptom during fever attacks in 12 TRAPS patients with AA amyloidosis [25]. Similarly, many studies confirm the association between cysteine substitutions and higher risk for amyloidosis [5, 26], while the detection of amyloidosis in patients with LP variants is highly unusual [3, 15, 25]. Nevertheless, since kidney amyloidosis has also been reported in R92Q and P46L patients, a regular screening of subclinical inflammation through the dosage of SAA levels remains recommended in patients carrying LP variants [7, 23, 27]. In this regard, the development of such unusual complication may depend on the presence of other concomitant genetic factors, such as different alleles of *SAA1* gene. Furthermore, the R92Q variant may act as an upregulator of the inflammatory response in a nonspecific manner, thus contributing to the likelihood of amyloidosis developing in certain patients affected by multifactorial chronic inflammatory disorders [28]. On this basis, the R92Q mutation is currently classified as a variant of uncertain significance, and its pathogenic role in causing TRAPS phenotypes is already controversial [29]. When considered pathogenic, it has been associated to a protean clinical phenotype consisting in fever, abdominal pain, arthralgia/arthritis, fatigue, myalgia, and less frequently headache, odynophagia, skin rash, and chest pain with a broad range of age at disease onset [3]. In line with previous evidences, in our cohort of patients carrying R92Q variant, disease onset was quite varied (ranging 1 to 54 years), with frequent flares lasting 12 days on average characterized by musculoskeletal symptoms, lymphadenopathy, skin rash, and chest pain as most frequently complained symptoms. Noteworthly, this subgroup of patients showed a slightly different clinical picture when compared to other genotypes, with a higher frequency of oral aphthosis and arthritis, lower occurrence of abdominal pain, and lower ESR levels during fever attacks. Patients with the R92Q variant were treated with NSAIDs monotherapy more frequently than others; nevertheless, more than half of them required biologic therapy in addition to NSAIDs, glucocorticoids, and/or colchicine along the course of the disease, disclosing a clinical profile of intermediate severity overall. It has been previously observed that patients carrying low-penetrance mutations may exhibit a stubborn clinical course, needing chronic administration of high doses of steroids and biologic therapies to achieve good disease control [30]. In this regard, the number of R92Q patients requiring cytokine blockers in our cohort is surprisingly higher than previously reported (2.9 to 22.2%) [2, 3, 7, 8, 15], although an omitted variable bias cannot be excluded: actually, the recently increased availability of biologic drugs and the greater familiarity of clinicians with their employment has brought about a widening of their use over the last years for both therapeutic and diagnostic purposes.

The recently developed and validated Eurofever classification score for TRAPS comprises two different sets of criteria: the first one is applicable to candidates carrying a confirming genotype or a variant of uncertain significance; the second one is designed to address appropriate patients towards genetic testing, or towards further genetic studies (search for somatic mosaicism); moreover, clinical criteria are formulated to classify patients for research purposes even in those countries where routine genetic testing is not possible. TRAPS classification criteria involving both genetic and clinical items displayed a sensitivity of 95% and a specificity of 99% when validated in the Eurofever cohort [9]. In our cohort, this set of criteria displayed a good performance (80% sensitivity overall) with a trend towards a better sensitivity in the pediatric setting than in the adult one, without reaching statistical significance. In addition, they provided high utility in classifying patients with HP variants (93.8% classified), while the sensitivity was lower in the LP group (77.3%) when using the diagnosis given by the treating physician as reference standard. These findings may be due to the relevant number of patients with low-penetrance mutations in our cohort, as negative or not confirmatory genotype has been associated to a minor classification capability of the new classification score [9]. Anyway, the inclusion of the genetic item (confirmatory and not confirmatory genotypes) has led to a better capability of classifying patients in all subgroups, especially among patients carrying LP variants.

On the other side, the version of clinical classification criteria not including genotype (*i.e.*, applicable prior to genetic analysis) displayed a sensitivity of 87% and a specificity of 92% when validated in the Eurofever cohort [9]. Nevertheless, in the present study, on the basis of clinical manifestations at disease onset, only 57.5% of patients resulted correctly classified as TRAPS. The performance was particularly unsatisfactory in the case of adult-onset disease (55.9% sensitivity) and among patients carrying LP variants (50%). It is worth mentioning that in the adult setting one out of five patients would have been classified also as FMF, while in the pediatric setting one out of five patients fulfilled MKD clinical classification criteria, disclosing a specificity of 76% and 80% for FMF criteria in adults and for MKD criteria in children, respectively. It is the authors' opinion that TRAPS clinical classification criteria can be conveniently used to address appropriate patients towards genetic testing or towards further in-depth genetic analysis, but their employment for classifying patients in countries where routine genetic testing is not available should be further assessed. Indeed, given the high phenotypic heterogeneity of the syndrome and the frequent overlap between TRAPS phenotype and other monogenic autoinflammatory diseases, a definite classification should rely on both accurate working clinical assessment and complementary genotype [31].

The multicentric retrospective nature of this study implies some limitations. Firstly, clinical data about disease onset in childhood were often recalled by adult patients; this retrospective collection might be determined some inaccuracy in some useful information could have been missed. Moreover, the majority of patients were Caucasian and our findings could not be applied directly and uncritically to patients from other geographic contexts. As regards the evaluation of classification criteria, two main limitations have been identified: firstly, the scores have been applied retrospectively on the basis of collected data, and it is not excluded some misinterpretation of symptoms; moreover, the absence of a control group has not allowed us to derive the specificity of the different sets of criteria. We outlined some clinical unmet needs in this field of research, which would be fruitfully addressed in the future: more detailed data about subsequent lines of biologic treatment are missing in the present study, as well as clear outcome measures when defining the response to treatments; moreover, predictive factors of response to different cytokines inhibition should be evaluated, with specific regard to genotypical asset; moreover, an extensive analysis of data about long-term follow-up of the pediatric-onset disease could allow the identification of different evolutive patterns and risk factors for severe outcome.

In conclusion, the present study describes one of the widest cohorts of TRAPS patients currently reported in the literature. Some genetic and clinical features, including the presence of mutations involving a cysteine residue, abdominal pain, and lymphadenopathy during attacks, significantly correlate with long-term and severe TRAPS-related complications. Our data confirm the high phenotypic heterogeneity of the disease, which seems more influenced by the penetrance of the mutation rather than by the age at onset itself, although clear genotype-phenotype associations still need to be defined.

## Figures and Tables

**Table 1:** Demographic characteristics of TRAPS patients described in the whole cohort of the study, according to age at onset and according to the penetrance of *TNFRSF1A* mutations. List of abbreviations: IQR: interquartile range; N.: number of patients; n.s.: not significant; SD: standard deviation; *TNFRSF1A*: tumor necrosis factor receptor super family member 1 A; TRAPS: tumor necrosis factor receptor-associated periodic syndrome.

	Whole *TRAPS* population	Pediatric onset group	Adult-onset group	*p*	High-penetrance *TNFRSF1A* mutations	Low-penetrance *TNFRSF1A* mutations	*p*
Numerosity N. (%)	80 (100%)	44 (55%)	36 (45%)		32 (40%)	44 (55%)	
Male : female	42 : 38	26 : 18	16 : 20	n.s.	19 : 13	20 : 24	n.s.
Age at recruitment, years Median (IQR) or mean ± SD	44.0 (31.0)	41.7 ± 19.8	40.4 ± 15.8	n.s.	48.7 ± 18.1	38.8 ± 16.5	0.04
Age at onset, years Median (IQR) or mean ± SD	8.0 (16.2)	4.9 ± 2.5	19.4 ± 2.5	<0.001	5.0 (14.1)	16.0 (26.5)	0.01
Age at diagnosis, years Mean ± SD	32.9 ± 15.9	28.3 ± 16.4	35.0 ± 13.7	0.003	39.6 ± 17.0	30.3 ± 14.5	0.01
Diagnostic delay, years Median (IQR) or mean ± SD	18.0 (27.5)	23.4 ± 16.0	15.6 ± 11.6	<0.001	30.0 (26.0)	7.0 (15.0)	<0.001
Duration of disease at last visit, years Median (IQR) or mean ± SD	26.0 (36.5)	33.8 ± 19.8	14.2 ± 10.3	0.002	35.5 (27.3)	12.0 (20.5)	0.002
Follow-up duration, years Median (IQR)	6.0 (8.0)	9.0 (8.5)	5.0 (14.0)	n.s.	8.0 (6.4)	5.0 (9.5)	n.s.

**Table 2:** Genotypical characterisation of TRAPS patients. List of abbreviations: *TNFRSF1A*: tumor necrosis factor receptor super family member 1 A; HGVS: Human Genome Variation Society.

	*TNFRSF1A* mutation(s)			Frequency
	HGVS sequence name	HGVS protein name	Usual name	
Low penetrance	c.123T>G	p.(Asp41Glu)	D12E	3
	c.143A>T	p.(Lys48Ile)	—	1
	c.224C>T	p.(Pro75Leu)	P46L	5
	c.362G>A	p.(Arg121Gln)	R92Q	32
	c.370G>A	p.(Val124Met)	V95M	1
	c.398G>A	p.(Arg133Gln)	R104Q	2

Unknown penetrance	c.1181G>A	p.(Arg394His)	—	1
	Others			3

High penetrance	c.214T>C	p.(Cys72Arg)	C43R	2
	c.215G>A	p.(Cys72Tyr)	C43Y	1
	c.236C>T	p.(Thr79Met)	T50M	10
	c.242G>A	p.(Cys81Tyr)	C52Y	3
	c.244A>G	p.(Arg82Gly)	R53G	2
	c.251G>A	p.(Cys84Tyr)	C55Y	1
	c.262T>C	p.(Ser88Pro)	S59P	2
	c.305G>A	p.(Cys102Tyr)	C73Y	2
	c.349T>G	p.(Cys117Gly)	C88G	1
	c.373T>C	p.(Cys125Arg)	C96R	3
	c.380G>T	p.(Cys127Phe)	—	1
	c.394_399del	p.(Tyr132_Arg133del)	Y103_R104DEL	1
	c.472+1G>A	p.(Cys158delinsYERSSPEAKPSPHPRG)	c.472+1G>A	2
	c.586_612del27	p.(Leu196_Gly204del)	L167_G175del	1

**Table 3:** Fulfillment of TRAPS Eurofever score according to Federici et al.. 2015 [13] (“TRAPS Eurofever score 2015”) and classification criteria according to Gattorno et al.. 2019 [9] (“genetic + clinical classificative criteria 2019” and “clinical classificative criteria 2019”) in the whole cohort of TRAPS patients and according to age at onset and *TNFRSF1A* mutations penetrance. List of abbreviations: CAPS: cryopyrin-associated periodic syndrome; FMF: familial Mediterranean fever; MKD: mevalonate kinases deficiency; n.a.: not available; n.s.: not significant; *TNFRSF1A*: tumor necrosis factor receptor super family member 1 A; TRAPS: tumor necrosis factor receptor-associated periodic syndrome.

	Whole TRAPS population	Pediatric-onset group	Adult-onset group	*p*	High-penetrance *TNFRSF1A* mutations	Low-penetrance *TNFRSF1A* mutations	*p*
TRAPS Eurofever score 2015 (%)	71.3	75.0	66.7	n.s.	93.8	56.8	<0.001
TRAPS genetic + clinical classificative criteria 2019 (%)	80.0	84.1	75.0	n.s.	93.8	77.3	n.s.
TRAPS clinical classificative criteria 2019 (%)	57.5	61.4	55.9	n.s.	71.9	50.0	n.s.
Fever ≥7days (%)	78.8	84.0	72.2	n.s.	87.5	79.5	n.s.
Fever 5–6 days (%)	8.8	13.6	2.8	n.s.	6.3	11.4	n.s.
Migratory rash (%)	6.3	4.5	8.3	n.s.	6.3	6.8	n.s.
Periorbital oedema (%)	17.5	22.7	11.1	n.s.	15.6	15.9	n.s.
Myalgia (%)	66.3	56.8	77.7	n.s.	56.3	72.7	n.s.
Positive family history (%)	38.8	52.3	22.2	0.01	56.3	25.0	0.01
Absence of aphthous stomatitis (%)	75.0	72.7	77.7	n.s.	90.6	68.2	0.02
Absence of pharyngotonsillitis (%)	70.0	65.9	75.0	n.s.	81.3	68.2	n.s.
FMF clinical classificative criteria 2019 (%)	12.5	4.5	23.5	0.01	12.5	11.9	n.s.
MKD clinical classificative criteria 2019 (%)	12.5	20.5	2.9	0.02	6.3	14.3	n.s.
CAPS clinical classificative criteria 2019 (%)	0	0	0	n.a.	0	0	n.a.
No clinical classificative criteria 2019 (%)	25	25	26.5	n.s.	15.6	33.3	n.s.
More than one clinical set of criteria 2019 (%)	11.3	13.6	8.8	n.s.	9.4	9.5	n.s.

## Data Availability

The datasets generated for this study are available on request to the corresponding author.
